# Formation and stability of oil-in-water nanoemulsions containing rice bran oil: *in vitro *and *in vivo *assessments

**DOI:** 10.1186/1477-3155-9-44

**Published:** 2011-09-28

**Authors:** Daniela S Bernardi, Tatiana A Pereira, Naira R Maciel, Josiane Bortoloto, Gisely S Viera, Gustavo C Oliveira, Pedro A Rocha-Filho

**Affiliations:** 1 Departamento de Ciências Farmacêuticas, Faculdade de Ciências Farmacêuticas de Ribeirão Preto, Universidade de São Paulo, Ribeirão Preto, São Paulo, Brazil; 2 Universidade de Ribeirão Preto, Ribeirão Preto, São Paulo, Brazil

## Abstract

**Background:**

Nanoemulsions have practical application in a multitude of commercial areas, such as the chemical, pharmaceutical and cosmetic industries. Cosmetic industries use rice bran oil in sunscreen formulations, anti ageing products and in treatments for skin diseases. The aim of this study was to create rice bran oil nanoemulsions using low energy emulsification methods and to evaluate their physical stability, irritation potential and moisturising activity on volunteers with normal and diseased skin types.

**Results:**

The nanoemulsion developed by this phase diagram method was composed of 10% rice bran oil, 10% surfactants sorbitan oleate/PEG-30 castor oil, 0.05% antioxidant and 0.50% preservatives formulated in distilled water. The nanoemulsion was stable over the time course of this study. *In vitro *assays showed that this formulation has a low irritation potential, and when applied to human skin during *in vivo *studies, the nanoemulsion improved the skin's moisture and maintained normal skin pH values.

**Conclusion:**

The results of irritation potential studies and *in vivo *assessments indicate that this nanoemulsion has potential to be a useful tool to treat skin diseases, such as atopic dermatitis and psoriasis.

## Background

Nanoemulsions are obtained when the size of an emulsion globule reaches approximately 20-500 nm. The small droplet size can resist the physical destabilisation caused by gravitational separation, flocculation and/or coalescence. It also avoids the creaming process because the droplet's Brownian motion is enough to overcome the gravitational separation force [1,2]. The size and polydispersity of nanoemulsions can affect properties such as particle stability, rheology, appearance, colour, texture and shelf life [3]. In nanoemulsions, the most frequent instability phenomenon is Ostwald ripening [4,5], which can be calculated according to the Lifshitz-Slezov and Wagner theory (LSW) using the following equation [6,7]:

(1) $$\omega =\frac{dr^{3}n}{dt}=k\frac{Dc_{\infty }\gamma M}{\rho^{2}RT}$$

Where *ω *is defined as the rate of change of the cube of the number average radius, *D *is the diffusion coefficient of the dispersed oil phase in the aqueous phase, γ is the interfacial tension between the two phases, *c*_∞_ is the bulk solubility of the oil in the water and ρ is the oil density. *k *is a constant that has the value of 8/9 in the LSW.

Nanoemulsions are well characterised and are a promising drug delivery system with practical applications for pharmaceutical, cosmetic and chemical industry applications. They have been used in intravenous, oral and ocular drug administrations and have reduced drug side effects and improved the pharmacological effects of the drugs given [8-10,4]. Nanoemulsions are primarily produced either by high-energy emulsification (e.g., high-pressure homogenisation) or by low-energy emulsification (using physicochemical properties of the components) [11]. This work focuses on the latter method for nanoemulsion synthesis.

Rice (*Oryza sativa*) bran oil has unsaponifiable fractions that contain high levels of antioxidant-rich components, such as tocopherols/tocotrienols and gamma-oryzanol, which could be useful for topical formulations [12]. The cosmetics industry has used rice bran oil in sunscreen formulations [13], in topical aging prevention products [14] and in treatments for skin diseases [15].

When skin is affected by diseases such as atopic dermatitis and psoriasis, it exhibits a compromised skin barrier function that causes increased transepidermal water loss [16-18]. Moisturisers can help improve the skin's function by relieving the cutaneous manifestations of these diseases [18-20]. Measuring the *stratum corneum *(SC) hydration degree gives important information about the biophysical properties and the function of the skin [21].

*In vitro *studies, such as the HET-CAM (Hen's Egg Test on the Chorioallantoic Membrane), are used to evaluate cosmetics products by immediately showing whether or not a solid or liquid substance irritates the hen's egg chorioallantoic membrane [22].

The aim of this study was to obtain nanoemulsions of rice bran oil and to evaluate their physical stability, irritating potential and *in vivo *moisturising activity.

## Materials and methods

### Materials

Sorbitan oleate (HLB 4.3) and rice bran oil were kindly provided from Lipo do Brasil (Brazil). PEG-30 castor oil (HLB 11.7) was donated by Oxiteno (Brazil). The preservative DMDM Hydantoin (and) Iodopropynyl Butylcarbamate (Glydant^® ^plus) was obtained from Chemyunion (Brazil), and the antioxidant Butyl Hydroxy Toluene (BHT) was purchased from Synth (Brazil).

### Preparation of nanoemulsion

The following surfactant mixtures were evaluated to find a stable nanoemulsion formulation: polysorbate 80/sorbitan oleate, polysorbate 60/sorbitan oleate, ceteareth-20 OE/ceteth-2, PEG-15 castor oil/sorbitan oleate, PEG-30 castor oil/sorbitan oleate and PEG-40 castor oil/sorbitan oleate. The PEG-30 castor oil/sorbitan oleate (HLB_resulting_= 8.0) was the only surfactant combination that resulted in a stable formulation and therefore was chosen for further study.

The phase diagram method was used with different concentration of constituents to result in 42 different emulsion formulations. All emulsions were prepared according the Emulsion Phase Inversion (EPI) method, where the water and oil phases were heated separately at 75°C, the water phase was added into the oil phase (rice bran oil and surfactants) while stirring at 600 rpm (Fisatom, 713-Dmodel, Brazil), and the mixture was then cooled to 25°C while stirring.

### Determination of nanoemulsion droplet size

The mean droplet size and polydispersity index of the nanoemulsions were determined by dynamic light scattering (DLS) (Zetasizer, modelo ZS, Malvern Instruments, UK). Measurements were performed at 25°C using a scattering angle of 90°. Samples were considered polydisperse when the polydispersity index was higher than 0.2 [23].

### Stability study

The preliminary stability of the nanoemulsion preparation was evaluated at 24 hours by centrifuge and thermal stress analyses. Stability was assessed by macroscopic emulsion observation and droplet size analysis. The purpose of these tests was to select a stable, low-surfactant formulation with a nanoemulsion-size droplet and stable physicochemical properties. The selected nanoemulsion was prepared in triplicate, and the samples were stored at 25 ± 2°C, 40 ± 2°C and 5 ± 2°C. Tests were performed at 24 hours, 7, 15, 30, 60 and 90 days after preparation. The analysis measurements were droplet size, pH value and electrical conductivity.

### Physical-chemical analyses

To perform the thermal stress test, nanoemulsions were heated in an ultra bath thermostat (Nova Técnica, Brazil) at temperatures ranging from 40 to 80°C. The temperature was increased by 5°C every 30 minutes. The nanoemulsions were centrifuged (Fanem 206-R, Brazil) at 1000, 2500 and 3500 rpm (70, 440 and 863 G, respectively) for 15 minutes in each rotation to accelerate possible instability phenomena. A pH meter (PM608 model - Analion, Brazil) was used to measure the pH of the nanoemulsions at 25 ± 2°C. The electrical conductivity was evaluated at 25 ± 2°C by a portable conductivity meter (mCA-150 model, Tecnopon, Brazil).

### Irritant test in an organotypic model - HET-CAM (Hen's Egg Test on the Chorioallantoic Membrane)

The HET-CAM test is routinely used to evaluate the potential eye irritation of raw materials but can in some cases be used to evaluate skin irritation, e.g. in the case of surfactants. Irritation causes alterations in the vascular system of the HET-CAM that result in membrane discoloration, haemorrhaging and increased perfusion. The method used in this manuscript is a modification of the method described by Luepke [24] and adapted by Mehling [25] that allows the immediate evaluation of irritation by solid or liquid substances in the hen's egg chorioallantoic membrane. Each substance was tested on three fertilised eggs that were incubated for 9 days prior to testing. The CAM (Chorioallantoic Membrane) was exposed to 300 μL of one of the following substances: (1) nanoemulsion (pH 6.54), (2) surfactant solution blend (5% sorbitan oleate, 5% PEG-30 castor oil and 90% water (pH 6.32), (3) Sodium lauryl sulphate (SLS) 10% w/w (positive control, pH 6.05) and (4) saline solution (negative control, pH 6.0). The CAM was rinsed with physiological saline solution after 30 seconds of exposure to each substance, and the intensity of the reactions (hyperaemia, haemorrhage and coagulation) was semi-quantitatively assessed on a scale of 0.5, 2 and 5 minutes after treatment; longer observation times give no additional important information. The numerical time-dependent scores for hyperaemia, haemorrhage and coagulation are summed to give a single numerical value indicating the irritation potential of the test substance on a scale with a maximum value of 21. The mean value of four tests makes possible an assessment by a classification scheme analogous to the Draize categories (Table 1).

**Table 1:** Classification of cumulative scores in the chorioallantoic membrane test (According Luepke 1985 [24])

Cumulative Score	Irritation assessment
0-0.9	Practically none
1-4.9	Slight
5-8.9	Moderate
9-21	Strong

### *In vivo *assessment

Approval to conducted the *in vivo *studies was obtained from the Ethics Committee of Faculdade de Ciências Farmacêuticas de Ribeirão Preto, Ribeirão Preto, São Paulo, Brazil, under protocol number CEP/FCFRP n°. 147.

Seventeen Caucasian volunteers with healthy skin (all women, 20-29 years old), five patients with atopic dermatitis (all women, 21-30 years old), and four psoriasis patients (1 man and 3 women, 28-56 years old) without skin lesions on the forearm were included in the study. The forearm area was washed with mild soap two hours before the analysis. The volunteers were kept for 10 min in a controlled room at ambient temperature (25 ± 2°C; 29-34% relative humidity) before starting the assay. Baseline values were determined using a Corneometer CM 820, a Sebumeter SM 810 and a Skin-pH-meter PH 900 instruments (Courage &Khazaka, Köln, Germany) before applying the formulation. Then, 50 μL of nanoemulsion was applied to the right forearm in three rectangular areas of 13.80 cm^2 ^and distributed for 20 seconds by rubbing the test area using a circular motion. Excess nanoemulsion was left on the skin. The treated skin of each patient was measured 30, 60, 90, 120 and 150 minutes after nanoemulsion application, with one measure for sebum content and triplicate measurements of electrical capacitance and skin pH.

## Results and discussion

### Preparation of nanoemulsion

**Figure 1:**
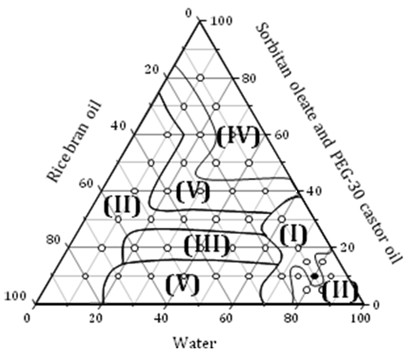
**Phase diagram with rice bran oil, sorbitan oleate/PEG-30 castor oil and water**. Region I: nanoemulsion, II: phase separation, III: gel phase, IV: W/O emulsions; V: O/W emulsions.

The phase diagram with the rice bran oil, surfactants sorbitan oleate/PEG-30 castor oil and water shows formation of five different areas: (I) O/W (Oil/Water) nanoemulsion, (II) phase separation, (III) gel phase, (IV) W/O (Water/Oil) emulsions and (V) O/W emulsions (Figure 1).

Table 2 shows the composition and the droplet size of the nanoemulsion systems.

**Table 2:** Composition of formulation characterised as nanoemulsions

Rice bran oil (% w/w)	Sorbitan oleate/PEG-30 castor oil (% w/w)	Purified water (% w/w)	Droplet size (nm) ± (Standard Deviation)
10.00	30.00	60.00	91 ± 19
10.00	20.00	70.00	45 ± 12
**10.00**	**10.00**	**80.00**	**69 ± 17**
20.00	10.00	70.00	303 ± 32
10.00	15.00	75.00	121 ± 13

All of the nanoemulsions (Table 2) were stable when tested using the centrifugation test. The only formulation that showed signs of instability at high temperatures (70°C) was the nanoemulsion composed of 10:20:70 (oil, surfactants and water, respectively).

A surfactant concentration of 5.00% was not sufficient to form a nanoemulsion, even with smaller amounts of oil. The surfactant amount affects the stabilisation and size of the emulsion droplets. From the experimental results, the nanoemulsion that used the lowest possible surfactant concentration while still maintaining thermal stability, centrifugal stability and small droplet size was selected as the working formulation.

Based on these preliminary results, we chose a formulation composed of 10% rice bran oil, 10% surfactant blend and 80% water and proceeded with further stability tests and *in vitro *and *in vivo *evaluations.

### Stability study

**Figure 2:**
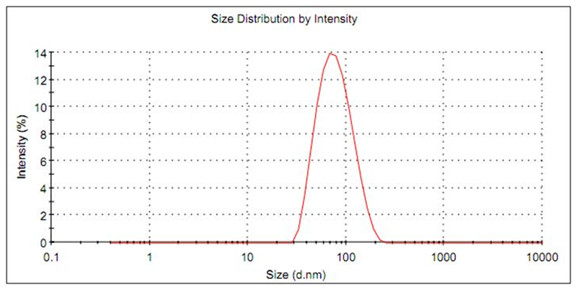
**Droplet size distribution of nanoemulsions after 24 hours**.

The formulation composed of 10:10:80 (rice bran oil, surfactant blend and water) was supplemented with 0.05% antioxidants and 0.50% preservatives. The formulation was tested at three different storage temperatures: 25 ± 2°C, 40 ± 2°C and 5 ± 2°C. By granulometric analysis the particles maintained a mono-disperse, monomodal peak after 24 hours (Figure 2). The formulation was stable for up to 90 days as determined by macroscopic analysis.

**Figure 3:**
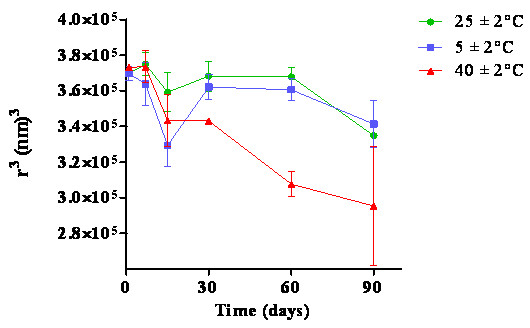
**Nanoemulsion droplet size under different storage conditions during a 90-day stability test**.

Droplet size measurements are a good indicator of the formulation stability. A fast droplet size increase indicates low system stability. The droplet size for this formulation remained constant over 90 days for all temperature conditions (Figure 3).

The nanoemulsions had polydispersity index values below 0.2 throughout the 90-day testing period, indicating the high fidelity of the system (low polydispersity), which may reflect the overall stability of this formulation and synthesis method. Polydispersity values near 1.0 are indicative of a polydisperse system [26]. The long term stability of nanoemulsions was previously evaluated and was also verified by stability studies conducted over three months. The W/O nanoemulsion produced by low energy emulsification showed no difference in droplet size over the study period at both 25°C and 4°C [27]. The W/O nanoemulsion demonstrated high physical stability, corroborating our results for temperatures of 5 ± 2°C and 25 ± 2°C.

Low-energy emulsification is better at producing stable nanoemulsions than its higher energy counterpart. When nanoemulsions were prepared using a high pressure homogeniser, the droplet size was initially around 100 nm; however, the particles increased in size after 30 days at either 25 or 4°C. This phenomenon was attributed to the preparation method [28]. The low-energy emulsification method used in our study showed high stability with respect to the droplet size and polydispersity index.

### pH value determination

Monitoring the pH value is important for determining the emulsions' stability because pH changes indicate the occurrence of chemical reactions that can compromise the quality of the final product. Emulsions produced with vegetable oils may experience a decrease in pH due to the hydrolysis of fatty acid esters into free fatty acid degradation products [29].

**Figure 4:**
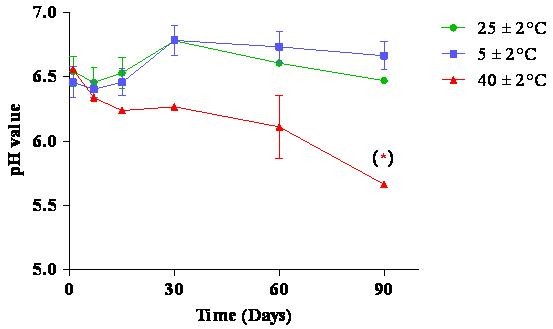
**pH values for nanoemulsions over time under different storage conditions**.

The nanoemulsions had stable pH values for almost all conditions tested (Figure 4). Only at a temperature of 40 ± 2°C and 90 days of incubation was there a statistically significant decrease in the pH of the nanoemulsion. The high temperature might have destabilised the nanoemulsion by hydrolysis, but it did not affect the overall quality of the nanoemulsions because the pH values remained around pH 6.0, which is an acceptable, non-skin irritating pH value.

### Electrical conductivity

**Figure 5:**
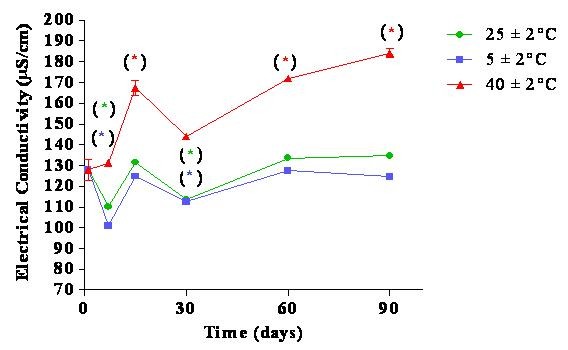
**Electrical conductivity of nanoemulsions over time under different storage conditions**.

The nanoemulsion showed changes in electrical conductivity at all storage conditions (Figure 5).

Changes in the electrical conductivity can indicate nanoemulsion instability and may influence the nanoemulsion droplet size [30]. In these studies, changes in electrical conductivity did not affect the nanoemulsion droplet size (Figure 3). It is difficult to assess the emulsion stability solely by electrical conductivity because the relationship between an increase in electrical conductivity and emulsion instability is not linear [31].

Thus, we could not conclusively determine the nanoemulsion's stability by this parameter. However, because the particle size and the pH value did not significantly change across different conditions, we considered our nanoemulsion to be stable. Nanoemulsion stability is a crucial parameter in determining the moisturising activity of the nanoemulsions *in vivo*.

### Irritant test in an organotypic model - HET-CAM (Hen's Egg Test on the Chorioallantoic Membrane)

Topical application products must have a low ocular/mucous membrane and a low dermal irritation potential. The irritation potential depends on the concentration of the substance as well as the chemical composition and the pH of the formulation [25].

The HET-CAM test can help evaluate the irritation potential of substances *in vitro *and *in vivo *[32]. The CAM showed no signs of irritation after application of either the nanoemulsion or the negative control substance, so the nanoemulsion was therefore considered practically non-irritating. The surfactant solution by itself caused mild hyperaemia, which suggests that the presence of rice bran oil in the nanoemulsion may have protected the chorioallantoic membrane from the irritating effects of the surfactant solution (Table 3). The pH values were the same for all samples tested to eliminate pH as a variable in the HET-CAM results.

**Table 3:** Scores and assessments of irritation potential of nanoemulsions and surfactant solution tested in the chorioallantoic membrane test

Formulation	Cumulative Score (average)	Assessment
Nanoemulsion^1^	0	Practically none
Surfactant Solution^2^	5	Moderate
Positive control^3^	15	Strong
Negative control^4^	0	Practically none

^1^10.00% of rice bran oil, 5.00% of sorbitan oleate, 5.00% of PEG-30 castor oil, 0.05% of BHT antioxidant, 0.50% of preservative and 79.45% of distilled water

^2^5.00% of sorbitan oleate, 5.00% of PEG-30 castor oil and 90.00% of distilled water

^3^10% of SLS and 90% of distilled water

^4^Saline solution

Previous studies showed that O/W microemulsions containing linoleic acid were only barely irritating in the HET-CAM test, indicated by a slight discoloration of the chorioallantoic membrane [33]. The HET-CAM test showed that the nanoemulsion containing rice bran oil was essentially non-irritating.

### *In vivo *assessment

The formulation composed of 10:10:80 (rice bran oil, surfactant blend and water) was chosen for the *in vivo *study due to its high stability and lack of irritation in the HET-CAM test. This nanoemulsion formulation was applied to volunteers with either normal or affected skin (atopic dermatitis or psoriasis).

### Moisturising activity

**Figure 6:**
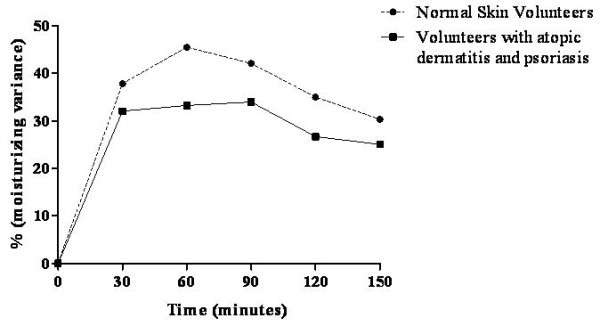
**Moisturising variance after nanoemulsion treatment in volunteers with either normal skin or skin affected by atopic dermatitis or psoriasis**.

The moisturising activity of the *stratum corneum *is measured by skin capacitance. It is also an important tool in evaluating healthy and diseased skin such as patients with atopic dermatitis or psoriasis [34-36]. The moisturising variance in healthy volunteers increased both 30 and 60 minutes after nanoemulsion application and then decreased over the remainder of the study. Volunteers with atopic dermatitis or psoriasis showed increased moisturising variance in the first 30 minutes and maintained this increase up to 90 minutes after application. Then, the moisturising capacitance decreased after 90 minutes until the end of test (Figure 6). Skin affected by atopic dermatitis or psoriasis had a lower basal hydration value compared with healthy skin. People with these drying skin conditions have increased dryness in their skin outside of the regions with lesions [37-39]. The rice bran oil nanoemulsion increased the moisturising variance by about 38% in normal skin volunteers and by 30% in volunteers with atopic dermatitis or psoriasis, which is a satisfactory result because a high-quality commercial moisturiser only increased skin hydration by about 20% after 14 days of application [40]. These improved effects may be caused by the nanoemulsion droplets adhering to the skin and forming a dense film that inhibits water evaporation from the skin [41].

The rice bran oil nanoemulsion significantly increased the skin hydration in volunteers suffering from atopic dermatitis and psoriasis. Although the skin hydration measurements should be conducted over a longer period of time, 8 - 24 h, this study indicates that the nanoemulsion may provide long-term skin hydration.

### Oily Skin

**Figure 7:**
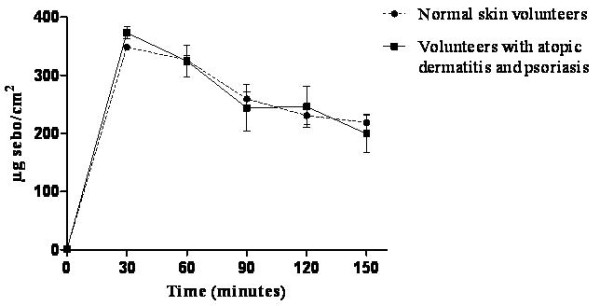
**Oiliness of the skin after nanoemulsion treatment in volunteers with either normal skin or skin affected by atopic dermatitis or psoriasis**.

The oiliness values of nanoemulsion-treated skin increased considerably 30 minutes after treatment and then decreased in both the healthy and affected skin groups. The increase may be related to the amount of rice bran oil (10%) in the formulation (Figure 7). Cosmetic emulsions form an oily layer on the skin that can protect the lipid barrier, which is desirable in dry skin conditions [42]. Therefore, the oiliness of the nanoemulsion may provide an alternative treatment for psoriasis.

### Skin pH determination

**Figure 8:**
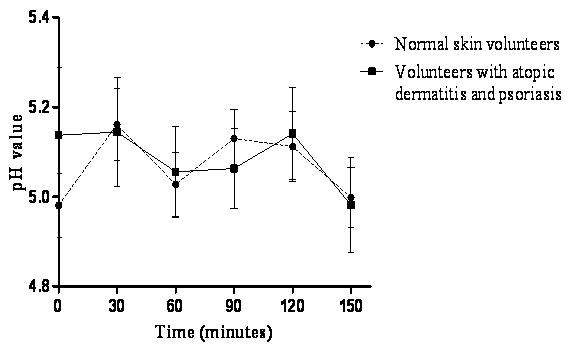
**Skin pH value after nanoemulsion treatment in volunteers with normal skin or skin affected by atopic dermatitis or psoriasis**.

Forearm skin testing is standard in most clinical studies of skin and has pH values in the range of 4.2 to 5.9 for both sexes [43]. The pH values of volunteers' skin tested during this study ranged from 4.9 to 5.2 after treatment for both groups. Thus, the pH changes due to the nanoemulsion were within the accepted pH range for forearm skin found in the literature, so this formulation does not significantly alter the skin pH (Figure 8).

## Conclusion

The nanoemulsion developed in this study using the phase diagram method was composed of 10% rice bran oil, 10% surfactants sorbitan oleate/PEG-30 castor oil, 0.05% antioxidants and 0.50% preservatives formulated in distilled water. The nanoemulsion was stable during the period of study and was found to be practically non-irritating in the organotypic HET-CAM model. When applied to the skin of volunteers, the nanoemulsion increased the relative hydration of the skin, the skin oiliness and maintained normal skin pH values. This nanoemulsion could serve as an alternative treatment for skin diseases such as atopic dermatitis and psoriasis.

## Competing interests

The authors declare that they have no competing interests.

## Authors' contributions

DSB selected the nanoemulsion composition through the phase diagram study. JB and DSB contributed to the stability study of formulation. NRM and DSB performed the irritant test in the organotypic model - HET-CAM. TAP and GSV performed the *in vivo *assessment. PARF and NRM guided the studies. The manuscript has been read and approved by all the authors.
